# Symptom response and episodic disability of long COVID in people with spinal cord injury: A case-control study

**DOI:** 10.1371/journal.pone.0304824

**Published:** 2024-06-28

**Authors:** Md. Feroz Kabir, Khin Nyein Yin, Ohnmar Htwe, Mohammad Saffree Jeffree, Fatimah Binti Ahmedy, Muhamad Faizal Zainudin, Sharmila Jahan, Md. Zahid Hossain, K. M. Amran Hossain, Md. Waliul Islam, Tofajjal Hossain

**Affiliations:** 1 Faculty of Medicine and Health Sciences, Universiti Malaysia Sabah, Kota Kinabalu, Sabah, Malaysia; 2 Department of Physiotherapy and Rehabilitation, Jashore University of Science and Technology (JUST), Jashore, Bangladesh; 3 Faculty of Medicine, University Kebangsaan Malaysia, Kuala Lumpur, Malaysia; 4 Faculty of Medicine, Universiti Teknologi MARA, Sungai Buloh, Malaysia; 5 Department of Physiotherapy, Centre for the Rehabilitation of the Paralysed (CRP), Savar, Dhaka, Bangladesh; The Hong Kong Polytechnic University, HONG KONG

## Abstract

**Background:**

Spinal cord injury (SCI) is a consequence of significant disability and health issues globally, and long COVID represents the symptoms of neuro-musculoskeletal, cardiovascular and respiratory complications.

**Purpose:**

This study aimed to identify the symptom responses and disease burden of long COVID in individuals with spinal cord injury.

**Methods:**

This case-control study was conducted on patients with SCI residing at a specialised rehabilitation centre in Bangladesh. Forty patients with SCI with and without long COVID symptoms (LCS) were enrolled in this study at a 1:1 ratio according to WHO criteria.

**Result:**

Twelve LCS were observed in patients with SCI, including fatigue, musculoskeletal pain, memory loss, headache, respiratory problems, anxiety, depression, insomnia, problem in ADL

problem in work, palpitation, and weakness. The predictors of developing long COVID include increasing age (p<0.002), increasing BMI (p<0.03), and longer duration of spinal cord injury (p<0.004). A significant difference (p<0.01) in overall years of healthy life lost due to disability (YLD) for non-long COVID cases was 2.04±0.596 compared to long COVID (LC) cases 1.22±2.09 was observed.

**Conclusion:**

Bangladeshi patients of SCI presented 12 long COVID symptoms and have a significant disease burden compared to non long COVID cases.

## Introduction

Long COVID (LC) or long COVID symptoms (LCS) can be defined as a persistent clinical symptoms that is manifest after 12 weeks of SARS-CoV-2 infection, persist for two months and cannot be explained by any other clinical diagnosis [1]. However, the symptoms can be episodic with relapsing-remitting symptoms that cause episodic disabilities [2]. Globally, 43% of post-COVID cases are prominent, and the rate was 50% in Asia [3]. Studies from Bangladesh revealed two important cohorts, reflecting that 16% to 24% of post-COVID-19 patients having long COVID [2, 4]. Considering the WHO clinical case definition globally, the prevalence of long COVID cases varies from 7% to 43% depending on the definition considered as long COVID [5]. In Bangladesh, another prevalence study showed that 22% of patients with LCS require rehabilitation services [6]. Moreover, according to couple of studies, the prevalence of LCS ranges from 71.21% to 77.7% [7, 8]. The most common symptom was respiratory manifestation, followed by fatigue at 51.4%. During the early phase of long COVID, fatigue, respiratory problems, headache, and joint pain were the prevalent symptoms [9].

Spinal cord injury (SCI) is a neurological disorder that occurs as a result of traumatic or pathological events and leads to substantial disability and health problems worldwide, including in Bangladesh [10]. With neurological deficits and spinal cord injury, people have challenges performing daily activities because of functional limitations, activity limitations, and physical, environmental, and socioeconomic barriers [11]. Approximately 19% of individuals who suffer from spinal cord injuries die within two years after being discharged from the hospital [12]. Cardiorespiratory involvement issues are a significant factor in predicting death for individuals with spinal cord injuries, along with pressure ulcers and other genitourinary complications [13]. Cardiorespiratory tract complications include cough, dyspnea, aspiration pneumonia, and severe respiratory infections [13]. Complications post-SCI are well-established, and these patients are vulnerable if they are infected by COVID-19, which commonly causes cardiovascular and respiratory complications.

In Bangladesh, cardiovascular and respiratory complications are common among patients with long COVID, and the presentations includ dyspnea, chest pain, fatigue, weakness, and palpitations being prominent symptoms [4, 5]. In addition, in spinal cord injury patients, there is a pre-existing comorbidity of respiratory complications [13]. Individuals with long COVID who have respiratory disorders, as well as those with spinal cord injuries, have a considerable burden of pre-existing cardiorespiratory and physical complications, in addition to the challenges posed by long COVID [5, 13]. People with more severe cervical SCI are more vulnerable to severe respiratory problems, and even in post-COVID-19, they had a risk of developing respiratory, immune, and other clinical complications that can lead to significant co-morbidities, mortality, and severe disease burden [14–16].

Since there are limited data on SCI patients with long COVID and disease burdens, a significant research gap is exsist both globally and in Southeast Asia. Previous scholarly literature has established a recommendation regarding the disease burden of people with vulnerable issues [5]. Disease burden can be expressed through disability-adjusted life years (DALYs), a time-based measure that combines the years of life lost due to premature disability, premature mortality (YLLs), and years of life lost due to time spent in the state of health that is less than complete or years of healthy life lost due to disability (YLDs). This study bridged a significant research gap by revealing the symptom response and episodic pattern of disability and its significant disease burden for long COVID in people with spinal cord injury. Hence, the objectives of this study were 1) to determine the symptom response of long COVID in people with spinal cord injury and their episodic disability and 2) to predict the factors contributing to a significant disease burden for long COVID in people with spinal cord injury.

## Methodology

### Study design

The study was a matched case-control study among patients with spinal cord injury who had long COVID and who resided at a specialised rehabilitation centre in Bangladesh [17].

### Sample size

We used Epi info software version 7.2.0 to calculate the sample size. We used matched pair case‒control study calculations. A total of 17.3% [18] of the SCI patients who had long COVID cases were exposed. A total of 22.4% of non-SCI patients with long COVID [6] were considered controls. In another study in the US [19], 6% of people with physical disability who recovered from COVID-19 were not exposed, and 14% of people with physical disabilities who had no COVID-19 were not exposed. The estimated OR was 0.2679 (95% CI; 0.1080, 0.6595). The McNemar test (χ2 9.4704, P<0.01) indicated a minimal sample size of 28 pairs. According to the specified timeline and from a population-based inception cohort [6], 28 patients with SCI and long COVID were screened between July and December 2021. The second screening was performed between July and August 2023. According to the WHO Working Group criteria, 20 of the screened patients had long COVID [1]. Therefore, our sample size was 20 pairs of cases and controls.

### Eligibility criteria

The primary eligibility criteria for the case were people with SCI aged more than 18 years with long COVID symptoms screened by a COVID-19 Yorkshire Rehabilitation Scale (C19-YRS). The eligibility for controls were people with SCI aged more than 18 years with no long COVID symptoms screened by a C19-YRS.

### Data collection

Data collection and clinical examination were performed by the trained researchers. Skilled researchers conducted the data collection process to ensure accuracy and consistency. Before data collection, practical training is provided to sound clinical screening and minimise potential bias. After that, during data collection, the researcher closely monitored the data collection process.

### Study procedure

**Fig 1:**
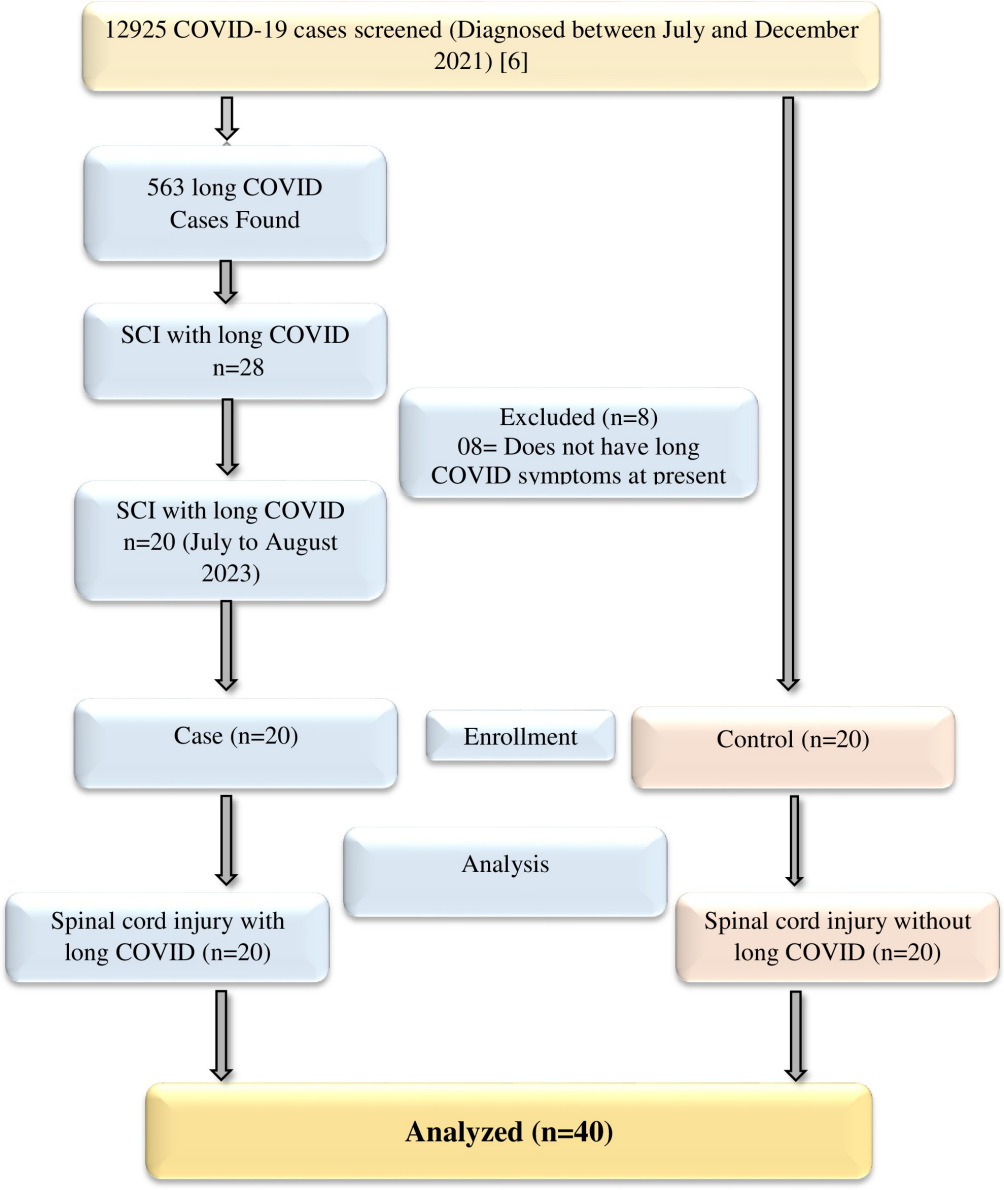
The flow chart of the study process.

To enhance the rigour of the study, we followed the Strengthening of the Reporting of the Observational Studies in Epidemiology (STROBE) guidelines for case-control studies. Fig 1 shows the flow diagram of the study process. The rehabilitation science researchers underwent a structured training program to become familiar with the diagnosis of long COVID symptoms before the study. Senior researchers constantly supervised by the study period.

### Tools

The neurological status of the patients with spinal cord injury was determined using the American Spinal Injury Association (ASIA) ASIA impairment scale (AIS), and their motor scores of key muscles, sensory scores, and total scores were recorded. The long COVID symptoms were diagnosed according to the C19-YRS, a 22-item questionnaire with definite answers. The questionnaire includes several symptoms to screen for long COVID cases, and the question is further sub-categorized based on symptom severity scores (0–100), functional disability scores (0–50), additional symptom scores (0–60), and overall health scores (0–10). The tool is valid and reliable for screening long COVID symptoms [20]. The disease burden was calculated according to disability-adjusted life years (DALYs), which was calculated by the sum of the years of life lost due to early death (YLLs) from that cause and the years of healthy life lost due to disability (YLDs) for individuals, not in a healthy as a result of the particular cause. The formula for YLLs is (the number of deaths) x L1 (life expectancy at death age), and the YLDs are calculated by the number of incidence cases for cause (I) x a disability weight (DW) x the average duration in years a person experiences with the case until recovery or death (L2) [21].

### Ethical consideration

Medical research ethics approval was obtained from the Medical and Research Ethics Committee, Universiti Malaysia Sabah (UMS/FPSK6.9/100-6/1/95), the Institute of Physiotherapy Rehabilitation, and the Research of Bangladesh Physiotherapy Association (BPA-IPRR/IRB/22/02/2023/078). The Clinical Trial Registry (CTRI)-India provided prospective trial registration (CTRI/2020/09/028165). The Helsinki Declaration was followed, and we obtained informed consent.

### Data analysis

The data was analysed using Statistical Package for Social Sciences version 20.0 (IBM SPSS 20.0). The significance level was set as an alpha value less than 0.05. Descriptive statistics were performed according to the nature of data for sociodemographic information, health-related information, spinal cord injury-related information, and long COVID-related information. The mean and standard deviation of the duration of symptoms have been expressed by weeks. The differences in parameters between case and control groups were compared and analysed by using independent t-test, chi-square, and Fisher exact test depending on the independent variable as bivariate, trivariate, and multivariate. The predictors of Long COVID cases that have been diagnosed have been determined through an unadjusted odds ratio with a 95% Confidence Interval (CI).

## Results

### Sociodemographic characteristics

The overall mean age of the respondents was 41±13.81 years, and that of the participants without long COVID symptoms was 33.05±10.9 years, with an LCS of 50.6±8.3 years. The majority of the patients (72.5%) were males (n = 29). Out of 40 participants, 21 individuals (52.5%) agreed to receive a third dose of COVID-19 vaccine. Among those who received the third dose, 11 out of 20 individuals without long COVID symptoms (55%) and 10 out of 20 participants with long COVID symptoms (50%) chose to be vaccinated again.

### Clinical presentation

The majority of respondents participating in the study were traumatic paraplegic 26 (65%), and non-traumatic paraplegic was 2(5%). The patients without LCS were both traumatic paraplegic and tetraplegic, 80% (16) of patients with LCS had non-traumatic paraplegic, and the rest of the patients were both traumatic tetraplegic and non-traumatic tetraplegic. The people with long COVID symptoms were diagnosed with AIS-B 5(25%), AIS-C 11(55%), and AIS-D 4(20%). The total motor score on the ASIA Impairment Scale was higher in people with long COVID symptoms 59.45±7.39 than those without long COVID symptoms 43.7±20.2. In the sensory score, people with LCS were also higher at 140±8.41 compared to those without long COVID symptoms at 116±5.7. The mean BMI score of the respondents was 26.48. The details of the socio-demographic parameters of people with or without LCS are presented in Table 1. There was a significant relationship between the AISA impairment scale and motor and sensory scores among the groups of people with and without LCS p<0.001.

**Table 1:** Presenting sociodemographic parameters with or without LCS.

	Overall		Participants without LCS		Participants with LCS		p-value
**General information**							
**Mean age (SD)**	41.83	(±13.081)	33.05	(±10.9)	50.6	(±8.3)	0.114^a^
**Gender**							
**Male (%)**	29	(72.5%)	12	(60%)	17	(85%)	0.155^b^
**Female (%)**	11	(27.5%)	8	(40%)	3	(15%)	
**COVID Vaccination**							
**1st dose**	3	(7.5%)	2	(10.0%)	1	(5.0%)	0.167^c^
**2nd dose**	10	(525%)	5	(25.0%)	5	(25.0%)	
**3rd dose**	21	(52.5%)	11	(55.0%)	10	(50.0%)	
**4th dose**	1	(2.5%)	1	(5.0%)	0	(0%)	
**No**	5	(12.5%)	1	(5.0%)	4	(20%)	
**Spinal Cord Injury-Related Information**							
**Diagnosis**							
**Traumatic paraplagic**	26	(65%)	10	(50%)	16	(80%)	0.290^c^
**Traumatic tetraplagic**	12	(30%)	10	(50%)	2	(10%)	
**Non-Traumatic paraplagic**	2	(5%)	0	(0%)	2	(10%)	
**ISNCSCI-Classification**							
**AIS-A**	7	(17.5%)	7	35%	0	(0%)	<0.001^c^
**AIS-B**	15	(37.5%)	10	50%	5	(25%)	
**AIS-C**	14	(35%)	3	15%	11	(55%)	
**AIS-D**	4	(10%)	0	(0%)	4	(20%)	
**Mean Total Motor score(SD)**	51.58	(±17.06)	43.7	(±20.295)	59.45	(±7.39)	0.002^a^
**Mean Total Sensory score(SD)**	128.38	(±42.26)	116.45	(±57.39)	140.3	(±8.49)	<0.001^a^
**Mean BMI(SD)**	26.48	(±0.52)	52.45	(±3.19)	69.55	(±5.28)	0.377^a^

^a^ Independent t-test

^b^ Chi-Squire

^c^ Fisher Exact test; LCS: Long COVID symptoms

### Long COVID symptoms in spinal cord injury patients

The long COVID symptoms in people with spinal cord injury had a diverse presentations. According to the COVID-19 Yorkshire Rehabilitation Scale, 12 symptoms have been observed in people with spinal cord injury having long COVID, including fatigue, musculoskeletal pain, memory loss, headache, respiratory problems, anxiety, depression, insomnia, palpitations, and weakness problems in the activity of daily living, and difficulty in work. The symptom of fatigue lasted for a more extended period of time (63.3±24 weeks), followed by musculoskeletal pain, which lasted for 57.6±16 weeks. Moreover, people with pre-existing depression, insomnia, problems with ADL, and problems in work long COVID had a more devastating effect. The people with long COVID experienced depression that lasted for more than 61.45±4.3 weeks, and limitations in the activity of daily living and participating at work lasted for 72±24 weeks, and 62.9±30 weeks respectively (Table 2).

**Table 2:** Long COVID symptoms in SCI duration are presented.

Symptoms	Mean duration in weeks	±SD
**Fatigue**	63.35	±24.8
**Musculoskeletal pain**	57.60	±16.9
**Memory**	36.50	±30.9
**Headache**	28.65	±33.8
**Respiratory Problem**	42.90	±37.9
**Anxiety**	51.50	±35.3
**Depression**	61.45	±24.3
**Insomnia**	63.35	±24.3
**Problem in ADL**	72.00	±24.8
**Problem in Work**	62.90	±30.1
**Palpitation**	45.75	±24.5
**Weakness**	20.15	±21.9

SCI: Spinal Cord Injury; ADL: Activity of Daily Living; SD: Standard Deviation

### Disability adjusted life years (DALYs)

**Fig 2:**
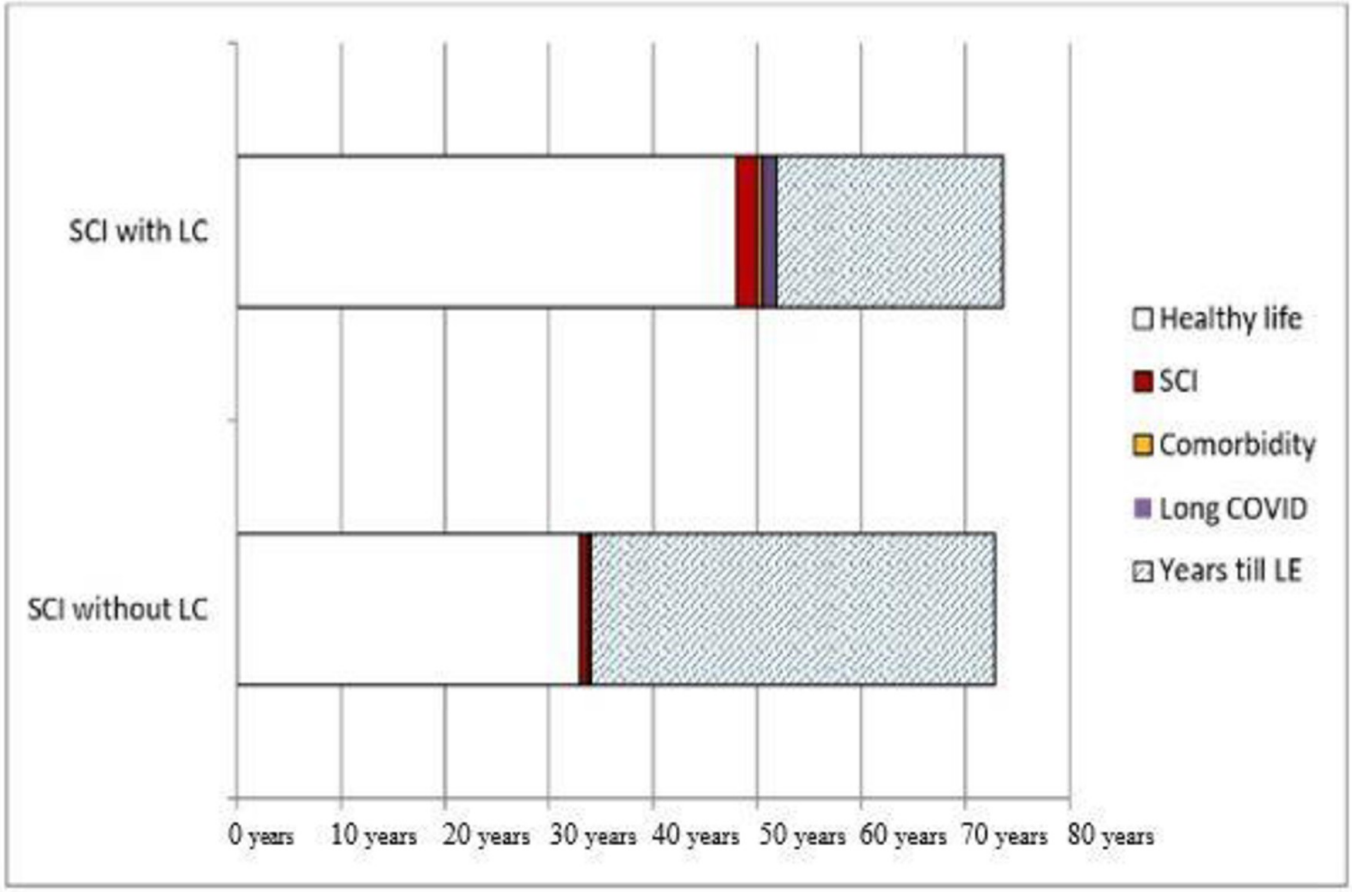
Presenting DALYs in long COVID and non-long COVID cases for people with SCI. Presenting mean duration in years; SCI: Spinal Cord Injury; LC: Long COVID; LE: Life Expectancy (72.3 years in Bangladesh).

The mean age at which people with SCI without long COVID had a healthy lifestyle was 33.05 years compared to people with long COVID as 50.6 years. The mean duration of SCI for non-long COVID patients was 0.75, and the mean duration of comorbidities was 0.25 years. For long COVID patients, the duration of SCI was two years, and the duration of comorbidity was 0.5 years, with a mean duration of long COVID of 1.4 years. The non-long COVID cases had 38.8 years till average life expectancy, and the long COVID patients had 21.7 years till average life expectancy. Fig 2 shows the DALYs in long COVID and non-long COVID cases. The YLL for non-long COVID patients was 38.9±10.9 years, and for long COVID patients, 21.4±8.2 years. A significant difference was observed in overall YLD for non-long COVID patients (2.04±0.596) compared to long COVID patients (1.22±2.09). DALYs in long COVID and non-long COVID patients for people with SCI are presented in Fig 2.

### Predictors of long COVID in spinal cord injury cases

Age was a significant (P<0.002) predictor for long COVID in spinal cord injury cases with an unadjusted odds ratio of 1.21 (95% CI;1.09–1.33), and BMI was another indicator (P = 0.03), unadjusted odds ratio of 1.76 (95% CI;1.04–2.09) (Table 3). The motor score of a spinal cord injury patient was also a significant predictor for long COVID with an unadjusted odd ratio 1.09 (95% CI;1.01–1.16), p = 0.013. The duration of spinal cord injury also predicted long COVID with an unadjusted odd ratio 1.06(95% CI;1.06–1.11), p = 0.004.

**Table 3:** Predictors of LC in SCI patients.

Dependent variable: long COVID		
**Independent variable**	Unadjusted OR(95%CI)	p-value
**Age**	1.21 (1.07, 1.33)	0.002**
**BMI**	1.76 (1.04, 2.97)	0.030*
**Motor score**	1.09 (1.01, 1.16)	0.013*
**Sensory score**	1.01 (0.998, 1.03)	0.080
**Duration of comorbidity**	4.77 (0.813, 28.01)	0.080
**Duration of SCI**	1.06 (1.02, 1.11)	0.004**
**YLD**	1.0 (0.116, 8.70)	0.990

* Significant with P<0.05

** significant with P<0.01; OR: Odds Ratio; CI: Confidential Interval; BMI: Body Mass Index; YLD: Years of healthy life lost due to disability; LC: Long COVID; SCI: Spinal Cord Injury

## Discussion

This groundbreaking study aimed to determine the symptom response and predictors of long COVID symptoms among vulnerable communities such as people with spinal cord injuries. In this matched case-control study design, 40 patients were selected; 20 participants with a spinal cord injury with long COVID symptoms and 20 without long COVID symptoms were determined and found that 12 of the symptoms were observed in SCI patients with long COVID and these findings were similar to those in the general population [4, 5]. The predictors of long COVID symptoms in patients with spinal cord injury were age, BMI, motor score, and duration of spinal cord injury. People with long COVID symptoms and spinal cord injury have a significant additional disease burden that hampers their memory, cardiorespiratory function, mental health issues, participation in daily living activities and work, and general lifestyle, which are common throughout the duration of the long COVID symptoms. This study answers the frequently asked questions about the vulnerability of long COVID in spinal cord injury patients to long COVID.

No similar case-control study has been conducted on patients with SCI with long COVID symptoms and their disease burdens. Previous studies on long COVID in the general population revealed that fatigue was one of the most important long COVID symptoms [3–6, 10]. The population proportion of fatigue ranges from 7% to 50% in the Asian region [17], and in Bangladesh, the percentage is 13% to 18% [4, 5]. In addition to fatigue, musculoskeletal pain is another long COVID symptom, with nearly 7% to 33% of the global and Asian people experiencing musculoskeletal pain [3], and in Bangladesh, the rate was nearly 10%. Interestingly, people having spinal cord injuries have multiple long COVID symptoms. Eight symptoms significantly impact multiple long COVID symptoms, such as fatigue, musculoskeletal pain, memory loss, headache, respiratory problems, anxiety, depression, insomnia, problems in ADL problems, work problems, palpitations, and weakness, each having multiple significant disease burdens [2–6, 10, 11]. Respiratory problems in patients with spinal cord injuries may worsen the respiratory symptoms of long COVID. This can result in longer recovery periods and increased use of healthcare services [22]. Another study noted that individuals with SCI have a higher risk of respiratory complications and increased respiratory symptoms associated with long COVID [23]. Additionally, individuals with SCI who already have cardiovascular health issues and compromised autonomic regulation may be at a greater risk of experiencing cardiovascular symptoms associated with long COVID [24]. In the general population, these long COVID symptoms had a significant impact on activity limitation, participation restriction, social participation, and environmental and personal factors in ICF domain. It was assumed that people with a spinal cord injury have an additional impact of long COVID in ICF domain [5].

The disease burden of a long COVID in patients with spinal cord injury is significant. Fig 1 presents a significant impact of the DALYs parameters in non-long COVID and long COVID symptoms for people with a spinal cord injury. Similar studies showed that long COVID can cause this additional disease burden according to DALY parameters [25, 26].

This study identified four predictors of long COVID spinal cord injury cases, including their age, body mass index, motor score, and the duration of spinal cord injury. The duration of spinal cord injury is among the important parameters that longer duration of spinal cord injury can increase the risk of developing long COVID. Similarly, with increasing age, increasing BMI and motor score, people with spinal cord injury can experience long COVID symptoms. The previous study in Bangladesh on the general population found [4] the predictors of long COVID gender, smoking, and severity of COVID as predictors. Another study [27] found that the predictors were subsequently similar. Still, people with set occupations, for example, a healthcare professional, a law enforcement agency, female, and older age have more chance of developing long COVID symptoms. In our study, age and BMI were matched with the population courses. Still, motor score and duration of spinal cord injury are new predictors of long COVID in spinal cord injury cases. A study found no increased mortality risk from COVID-19 among those with a history of SCI, but diagnosis-associated LCS symptoms may worsen disability after infection [28]. It is necessary to establish a long COVID rehabilitation protocol for vulnerable people with significant neurological issues, including spinal cord injury, and to monitor the additional disease burden of long COVID in spinal cord injury cases as a routine protocol.

### Strength and limitation

The strength of this study was exploring a recommended research question that can significantly impact long COVID rehabilitation, especially in spinal cord injury cases. The study had some limitations, such as a small sample population from the largest rehabilitation hospital in Bangladesh. The reasons are stringent inclusion and exclusion criteria with fewer patients with SCI with long COVID symptoms. This is because there has no clearcut statement of the prognosis of SCI with long COVID. Each patient has a different prognosis and outcome. Therefore, the inclusion and exclusion criteria confirmed, we extracted the maximum number of homogeneous data from the population. However, the sample size with 20 people in each group was sufficient for a significant effect. We overcame the limitation by collaborating with patients in the organisation and strictly following the WHO Working Group criteria while recruiting cases from skilled and experienced healthcare professionals to ensure valid and justified answers to scientific queries. We recommend further study on the long-term consequences of long COVID and its impact on survivor-ship in spinal cord injury patients.

## Conclusion

Twelve long COVID symptoms were observed in individuals with Spinal Cord Injury (SCI) in Bangladesh. The most prominent adverse effects on individuals with SCI and long COVID symptoms included fatigue, neuro-musculoskeletal discomfort, mental health issues, and limits in physical activity. Factors associated with the development of long COVID were advanced age, elevated BMI, and a prolonged period of spinal cord damage. Bangladeshi individuals with a spinal cord injury who had LCS experienced a substantial disease burden in comparison of non-long COVID cases, necessitating additional medical attention.

## Abbreviations

ADL: Activity of Daily Living; BMI: Body Mass Index
CI: Confidential Interval
C19-YRS: COVID‐19 Yorkshire Rehabilitation Scale
ICF: International Classification of Functioning, Disability and Health
LCS: Long COVID symptoms
SCI: Spinal Cord Injury
YLDs: Years of healthy life lost due to disability
YLLs: Years of life lost

## References

[1] World Health Organization. A clinical case definition of post covid-19 condition by a Delphi Consensus, 6 October 2021. 1970. https://apps.who.int/iris/handle/10665/345824

[2] O’Brien KK, Brown DA, McDuff K, Clair-Sullivan NS, Solomon P, Carusone SC, et al. Conceptualising the episodic nature of disability among adults living with Long COVID: a qualitative study. BMJ global health. 2023 Mar 1;8(3):e011276. doi: 10.1136/bmjgh-2022-011276 36863719 PMC9979585

[3] Chen C, Haupert SR, Zimmermann L, Shi X, Fritsche LG, Mukherjee B. Global prevalence of post-coronavirus disease 2019 (COVID-19) condition or Long covid: A meta-analysis and systematic review. The Journal of Infectious Diseases. 2022;226(9):1593–1607. 10.1093/infdis/jiac136 35429399 PMC9047189

[4] Hossain MA, Hossain KM, Saunders K, et al. Prevalence of Long covid symptoms in Bangladesh: A prospective inception cohort study of covid-19 survivors. BMJ Global Health. 2021;6(12). 10.1136/bmjgh-2021-006838 34906986 PMC8671853

[5] Chakrovorty SK, Hossain KA, Shafin R, Ahammad S, Jahid IK. Does post-COVID-19 characterize new diseases and disabilities?. The Lancet Regional Health-Southeast Asia. 2023 Aug 1;15. 10.1016/j.lansea.2023.100234 37305608 PMC10234345

[6] Kabir M.F., Yin K.N., Jeffree M.S. et al. Profile of long COVID symptoms needing rehabilitation: a cross-sectional household survey of 12,925 SARS-CoV-2 cases between July and December 2021 in Bangladesh. *Arch Public Health* 81, 132 (2023). 10.1186/s13690-023-01140-0 PMC10351147 37461092

[7] Kim Y, Bae S, Chang HH, Kim SW. Long COVID prevalence and impact on quality of life 2 years after acute COVID-19. Scientific Reports. 2023 Jul 11;13(1):11207. https://doi.org/10.1038%2Fs41598-023-36995-4 37433819 10.1038/s41598-023-36995-4 PMC10336045

[8] Jangnin R, Ritruangroj W, Kittisupkajorn S, Sukeiam P, Inchai J, Maneeton B, et al. Long-COVID Prevalence and Its Association with Health Outcomes in the Post-Vaccine and Antiviral-Availability Era. Journal of Clinical Medicine. 2024 Feb 21;13(5):1208. doi: 10.3390/jcm13051208 38592016 PMC10931928

[9] Takakura K, Suka M, Kajihara M, Koido S. Clinical features, therapeutic outcomes, and recovery period of long COVID. Journal of Medical Virology. 2023 Jan;95(1):e28316. doi: 10.1002/jmv.28316 36412057 PMC10108287

[10] Anjum A, Yazid MD, Fauzi Daud M, Idris J, Ng AM, Selvi Naicker A, et al. Spinal cord injury: pathophysiology, multimolecular interactions, and underlying recovery mechanisms. International journal of molecular sciences. 2020 Oct 13;21(20):7533. doi: 10.3390/ijms21207533 33066029 PMC7589539

[11] Savic G, Frankel HL, Jamous MA, Soni BM, Charlifue S. Participation restriction and assistance needs in people with spinal cord injuries of more than 40 year duration. Spinal cord series and cases. 2018 Mar 27;4(1):28. doi: 10.1038/s41394-018-0056-9 29619249 PMC5871617

[12] White JL, Sheth KN, editors. Neurocritical care for the advanced practice clinician. Springer International Publishing; 2018.

[13] Sezer N, Akkuş S, Uğurlu FG. Chronic complications of spinal cord injury. World journal of orthopedics. 2015 Jan 1;6(1):24. doi: 10.5312/wjo.v6.i1.24 25621208 PMC4303787

[14] Berlowitz DJ, Wadsworth B, Ross J. Respiratory problems and management in people with spinal cord injury. Breathe. 2016 Dec 1;12(4):328–40. doi: 10.1183/20734735.012616 28270863 PMC5335574

[15] Soriano JB, Kendrick PJ, Paulson KR, Gupta V, Abrams EM, Adedoyin RA, et al. Prevalence and attributable health burden of chronic respiratory diseases, 1990–2017: a systematic analysis for the Global Burden of Disease Study 2017. The Lancet Respiratory Medicine. 2020 Jun 1;8(6):585–96. doi: 10.1016/S2213-2600(20)30105-3 32526187 PMC7284317

[16] Galeiras Vázquez R, Rascado Sedes P, Mourelo Fariña M, Montoto Marqués A, Ferreiro Velasco ME. Respiratory management in the patient with spinal cord injury. BioMed research international. 2013 Jan 1;2013. doi: 10.1155/2013/168757 24089664 PMC3781830

[17] CRP. Available from: https://www.crp-bangladesh.org/

[18] Abramoff BA, Hentschel C, Dillingham IA, Dillingham T, Baraniecki‐Zwil G, Williams A, et al. The Association of Multiple Sclerosis, Traumatic Brain Injury, and Spinal Cord Injury to Acute and Long COVID‐19 Outcomes. PM&R. 2023 Dec 25. 10.1002/pmrj.13121 PMC11189756 38145343

[19] Lau B, Wentz E, Ni Z, Yenokyan K, Coggiano C, Mehta SH, et al. Physical and mental health disability associated with long-COVID: Baseline results from a US nationwide cohort. medRxiv. 2022 Dec 7.doi: 10.1101/2022.12.07.22283203 PMC10924070 37690503

[20] Hossain MS, Harvey LA, Islam MS, Rahman MA, Liu H, Herbert RD, et al. Loss of work-related income impoverishes people with SCI and their families in Bangladesh. Spinal Cord. 2020 Apr;58(4):423–9. doi: 10.1038/s41393-019-0382-1 31772346 PMC7138756

[21] Straudi S, Manfredini F, Baroni A, Milani G, Fregna G, Schincaglia N, et al. Construct validity and responsiveness of the COVID-19 Yorkshire Rehabilitation Scale (C19-YRS) in a cohort of Italian hospitalized COVID-19 patients. International Journal of Environmental Research and Public Health. 2022 May 30;19(11):6696. doi: 10.3390/ijerph19116696 35682280 PMC9180312

[22] Struijk EA, May AM, Beulens JW, de Wit GA, Boer JM, Onland-Moret NC, et al. Development of methodology for disability-adjusted life years (DALYs) calculation based on real-life data. PLoS One. 2013 Sep 20;8(9):e74294. doi: 10.1371/journal.pone.0074294 24073206 PMC3779209

[23] Jones R, Davis A, Stanley B, Julious S, Ryan D, Jackson DJ, et al. Risk predictors and symptom features of long COVID within a broad primary care patient population including both tested and untested patients. Pragmatic and observational research. 2021 Aug 11:93–104. doi: 10.2147/POR.S316186 34408531 PMC8366779

[24] Eriks-Hoogland IE, Barth MA, Müller LL, Braun D, Curt A, Arora M, et al. COVID-19 and spinal cord injury: clinical presentation, clinical course, and clinical outcomes of people hospitalised. Spinal cord series and cases. 2024 Feb 13;10(1):5. doi: 10.1038/s41394-024-00617-6 38351025 PMC10864293

[25] Carfì A, Bernabei R, Landi F. Persistent symptoms in patients after acute COVID-19. Jama. 2020 Aug 11;324(6):603–5. doi: 10.1001/jama.2020.12603 32644129 PMC7349096

[26] Howe S, Szanyi J, Blakely T. The health impact of long COVID during the 2021–2022 Omicron wave in Australia: a quantitative burden of disease study. International Journal of Epidemiology. 2023 Apr 3:dyad033. 10.1093/ije/dyad033 PMC10244043 37011639

[27] Bowe B, Xie Y, Al-Aly Z. Postacute sequelae of COVID-19 at 2 years. Nature medicine. 2023 Aug 21:1–1. doi: 10.1038/s41591-023-02521-2 37605079 PMC10504070

[28] Chakrovorty SK, Hossain KM, Hossain MA, Ahammad S, Kabir M, Shafin R, et al. Predictors of and Factors Associated with Novel Post COVID Symptoms in the Musculoskeletal, Functional, and Cognitive Domains for Vaccinated Delta-Variant Survivors: A Descriptive Survey of a Nationwide Prospective Inception Cohort in Bangladesh. 10.2139/ssrn.4249920

